# Amino acid substitutions in CPC-LIKE MYB reveal residues important for protein stability in *Arabidopsis* roots

**DOI:** 10.1371/journal.pone.0205522

**Published:** 2018-10-11

**Authors:** Koh Yamada, Michiko Sasabe, Yukichi Fujikawa, Takuji Wada, Rumi Tominaga-Wada

**Affiliations:** 1 School of Applied Biological Science, Hiroshima University, Higashi-Hiroshima, Japan; 2 Faculty of Agriculture and Life Science, Hirosaki University, Hirosaki, Japan; 3 Graduate School of Biosphere Science, Hiroshima University, Higashi-Hiroshima, Japan; Indiana University, UNITED STATES

## Abstract

*TRYPTICHON* (*TRY*) and *ENHANCER OF TRY AND CPC2* (*ETC2*) encode R3-type MYB transcription factors that are involved in epidermal cell differentiation in *Arabidopsis thaliana*. *TRY* and *ETC2* belong to the *CPC-like MYB* gene family, which includes seven homolog genes. Previously, we showed that among the CPC family members, TRY and ETC2 are characterized by rapid proteolysis compared with that of other members, and we demonstrated that this proteolysis is mediated by the proteasome-dependent pathway. In this study, we compared the functions of the wild-type TRY and ETC2 proteins and their amino acid-substituted versions. Our results showed that the substitution of amino acids in the C-terminal of TRY and ETC2 conferred them the ability to induce root hair formation. Furthermore, we confirmed that these mutations enhanced the stability of the TRY and ETC2 proteins. These results revealed that the amino acids, which are important for the functions of TRY and ETC2, mediate morphological pattern formation and can be useful in understanding the pathway determining the fate of root hair cells.

## Introduction

The formation of root hair and non-hair cell is a well-studied model system of pattern formation in *Arabidopsis thaliana* [1, 2]. A transcription factor complex comprising WEREWOLF (WER) [3], TRANSPARENT TESTA GLABRA (TTG1) [4], and GLABRA3/ENHANCER OF GL3 (GL3/EGL3) [5, 6] promotes the expression of the downstream *GLABRA2* gene (*GL2*) [7], and the expression of *GL2* results in non-hair cell formation [8, 9]. On the contrary, the fate of root hair cell is decided by the *CAPRICE* gene (*CPC*), which encodes an R3-type MYB transcription factor [10]. *CPC* has six additional homologs in the *Arabidopsis* genome, including *TRY*, *ENHANCER OF TRY AND CPC1* (*ETC1*), *ETC2*, *ETC3/CPC LIKE MYB3* (*CPL3*), *TRICHOMELESS1* (*TCL1*), and *TCL2* [11–18]. Basically, these seven *CPC* family genes are believed to act as inducers of root hair differentiation and repressors of trichome formation [9, 18]. Although, the *CPC* family genes have common characteristics in regulating the root hair and trichome development as described above, their specific functions differ [19].

Previously, to understand the precise functions of the *CPC* family members in regulating the root hair and trichome development, we observed the phenotypes of transgenic plants expressing *CPC*, *TRY*, *ETC1*, *ETC2*, and *CPL3* under a *CPC* promoter, and analyzed the localization of the encoded proteins in *Arabidopsis* root epidermis [20]. Compared with that of the wild type plants, the transgenic plants expressing *CPC*, *ETC1*, and *CPL3* showed a significant increase in the number of root hairs. In contrast, transgenic plants expressing *TRY* and *ETC2* did not show any obvious induction of ectopic root hairs [20]. TRY and ETC2 have an extended C-terminal region, unlike that of CPC, ETC1, and CPL3. Therefore, we deleted 19 and 18 amino acid regions from the C-termini of TRY and ETC2, respectively, and observed that these deletions resulted in TRY and ETC2 having functions similar to those of CPC, ETC1, and CPL3 [20]. We also demonstrated that the deletion of C-termini of TRY and ETC2 contributed to the stability of these proteins [20].

In this study, we focused on the C-terminal region of TRY and ETC2. To better understand the precise functions of approximately 20 amino acids in the C-terminal region of TRY and ETC2, we conducted phenotypic analyses of plants expressing amino acid-substituted TRY and ETC2 and assessed the localization and stability of the mutated proteins.

## Materials and methods

### Gene constructs

**Fig 1:**
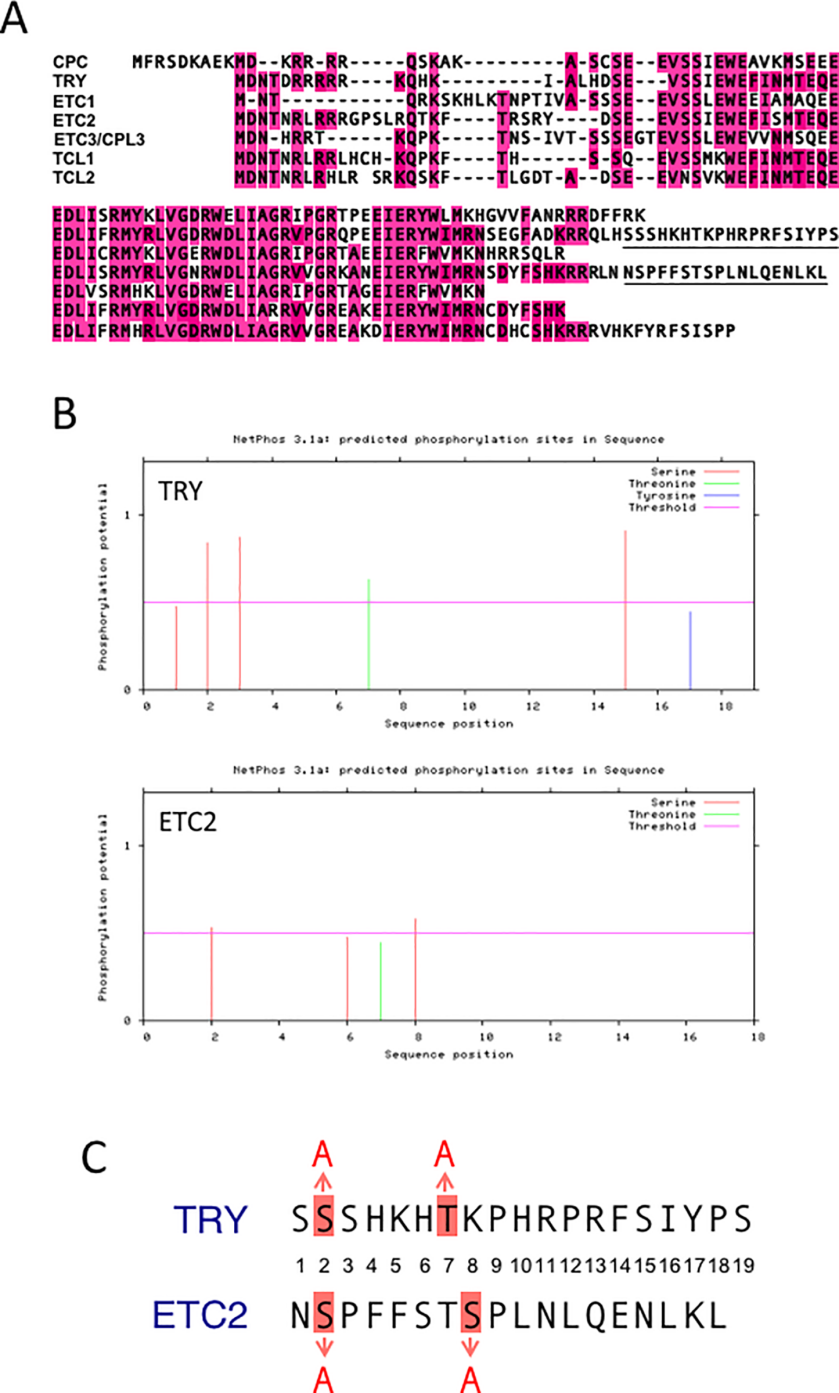
Amino acid substitution in TRY and ETC2. (A) Sequence alignment of CPC family MYB proteins. Amino acids displaying high homology are shaded in pink. The extended C termini of TRY and ETC2 are underlined. (B) Putative phosphorylation sites of TRY C-terminal region (upper) and ETC2 C-terminal regions (lower) predicted using NetPhos 3.1. (C) Alignment of the C-terminal region of TRY and ETC2. Numbers indicate the position from the N-terminus of the C-terminal sequences of TRY and ETC2. Amino acids substituted by A are indicated and shaded in red.

***CPC*::*TRY(SATA)*:*GFP*.** The *CPC*::*TRY(SATA)*:*GFP* construct was generated with the *CPC*::*TRY*:*GFP* backbone [20] by TaKaRa (TaKaRa, Kyoto, Japan). To create the *CPC*::*TRY(SATA)*:*GFP* construct, the *TRY*-specific DNA sequence corresponding to S and T amino acids (S2 and T7) in the C-terminal region of TRY was substituted by the codon for A (Fig 1C).

***CPC*::*ETC2(SASA)*:*GFP*.** The *CPC*::*ETC2(SASA)*:*GFP* construct was generated with the *CPC*::*ETC2*:*GFP* backbone [20] by TaKaRa (TaKaRa). To create the *CPC*::*ETC2(SASA)*:*GFP* construct, the *ETC2*-specific DNA sequence corresponding to two S amino acids (S2 and S8) in the C-terminal region of ETC2 was substituted by the codon for A (Fig 1C).

### Plant materials and growth conditions

In this study, *Arabidopsis thaliana* (L.) Heynh ecotype Columbia (Col-0) was used as the wild type plant. The seeds were surface sterilized and seeded on 1.5% agar plate as described before [21]. After seeding, the plates were maintained at 4°C for 2 d and incubated at 22°C under constant white light (50–100 μmol m^-2^s^-1^). For each transgenic line, five 10-day-old seedlings were used to count the number of root hairs and five 2-week-old third leaves were used to count the number of trichomes.

### Transgenic plants

The floral dip method was used for plant transformation [22], and the transgenic plants were selected on 0.5× Murashige and Skoog’s (MS) agar plates containing 50 mg/L kanamycin. The homozygous transgenic lines were selected for kanamycin resistance. At least 12 T1 lines were isolated for each construct and at least six T2 and three T3 lines were selected based on their segregation ratios for kanamycin resistance.

### Analysis of the phosphorylation sites

To analyze the potential phosphorylation sites in the C-terminal region of TRY and ETC2, the amino acid sequences of TRY (SSSHKHTKPHRPRFSIYPS) and ETC2 (NSPFFSTSPLNLQENLKL) were submitted to the NetPhos 3.1 (http://www.cbs.dtu.dk/services/NetPhos/) and PhosPhAt (http://phosphat.uni-hohenheim.de/) online tools [23, 24].

### Real-time reverse transcription PCR analysis

The total RNA was extracted from the roots and used for real-time RT-PCR analysis, as described previously [25], using primer pairs for *TRY*, *ETC2*, and *ACT2*, as described previously [20]. The relative expression of each transcript was determined by the ΔΔCt method [26]. *ACT2* was used as an endogenous control to normalize the expression level of *TRY* and *ETC2*.

### Immunoblot analysis

The proteins were extracted from the whole-cell-extracts of root tissue using the P-PER Plant Protein Extraction Kit (Thermo Scientific, MA, USA), according to the manufacturer's instructions. The extracted proteins (20 μg) were separated by SDS-PAGE on a 10% Mini-PROTEAN EGX Precast Gel (Bio-Rad, CA, USA), and were then transferred onto a PVDF membrane (Bio-Rad). We used mouse anti-GFP antibody (1:10000; Living Colors A.v. Monoclonal Antibody; Clontech, CA, USA) and HRP-linked sheep anti-mouse IgG antibody (1:10000; Amersham ECL Anti-Mouse IgG HRP-Linked Species-Specific Whole Antibody from sheep; GE Healthcare, Little Chalfont, UK) for western blotting. The immunoblotted proteins were detected with the ImmunoStar LD (Wako, Osaka, Japan) and Ez-Capture MG imaging systems (ATTO, Tokyo, Japan).

### Microscopy

#### Light microscopy

For each transgenic line, 10-day-old seedlings were analyzed to determine the number of root hairs by light microscopy using a Leica MZ16FA stereomicroscope (Leica Microsystems GmbH, Wetzlar, Germany). The images were recorded using a high-sensitivity CCD color camera system (Keyence VB 7010, Osaka, Japan). To analyze the trichomes, the images were captured using a VC4500 3D digital fine microscope (Omron, Kyoto, Japan) or a digital microscope (VH-700; Keyence).

#### Confocal laser scanning microscopy

The transgenic GFP fusion lines were stained with 5 μg/mL propidium iodide (PI) for 10 s, and then washed with water. The confocal images were obtained with a Zeiss LSM-700 Meta confocal laser scanning microscope (CLSM) using 488-nm laser lines for GFP excitation. Image processing was performed with Photoshop version 7.0 (Adobe Systems, CA, USA).

## Results

### Amino acid substitution in the C-terminal regions of TRY and ETC2

The CPC family genes encode homologous R3 type MYB transcription factor proteins (Fig 1A). Among them, only TRY and ETC2 have extended C-terminal sequences (Fig 1A). Previously, we demonstrated that these C-terminal regions are responsible for rapid degradation of the TRY and ETC2 proteins [20]. In this study, to narrow down the residues that are important for protein function and stability in the extended C-terminal regions of TRY and ETC2, we performed sequence analysis on these regions using the known consensus motifs (Fig 1). The C-terminal amino acid sequences of TRY and ETC2 did not have any common amino acid motif. In addition, we did not detect any possible PEST sequences in either TRY or ETC2; these sequences are known to target themselves for proteolytic degradation [27]. Phosphorylation is a fundamental mechanism through which protein function is regulated [28]. Therefore, we searched for candidate phosphorylation sites in the C-terminal regions of TRY and ETC2. The phosphorylation site analysis (using NetPhos) showed the presence of putative phosphorylation sites in the C-terminal sequences of TRY (S2, S3, T7, and S15) and ETC2 (S2 and S8) (Fig 1B). Contrarily, phosphorylation site analysis with PhosPhAt showed putative phosphorylation sites in the C-terminal sequences of TRY (T7) and ETC2 (S2 and S8) (S1 Fig). PhosPhAt analysis detected only T7 as a phosphorylation site in the C-terminal sequences of TRY (Figure A in S1 File). However, the amino acid alignment showed that S2 of TRY corresponds to S2 of ETC2. Based on these results of NetPhos and PhosPhAt analyses, we selected the amino acids, S2 and T7 in TRY, and S2 and S8 in ETC2, as the amino acid substitution sites (Fig 1C).

### Amino acid substitutions confer root hair inducing function on TRY and ETC2

**Fig 2:**
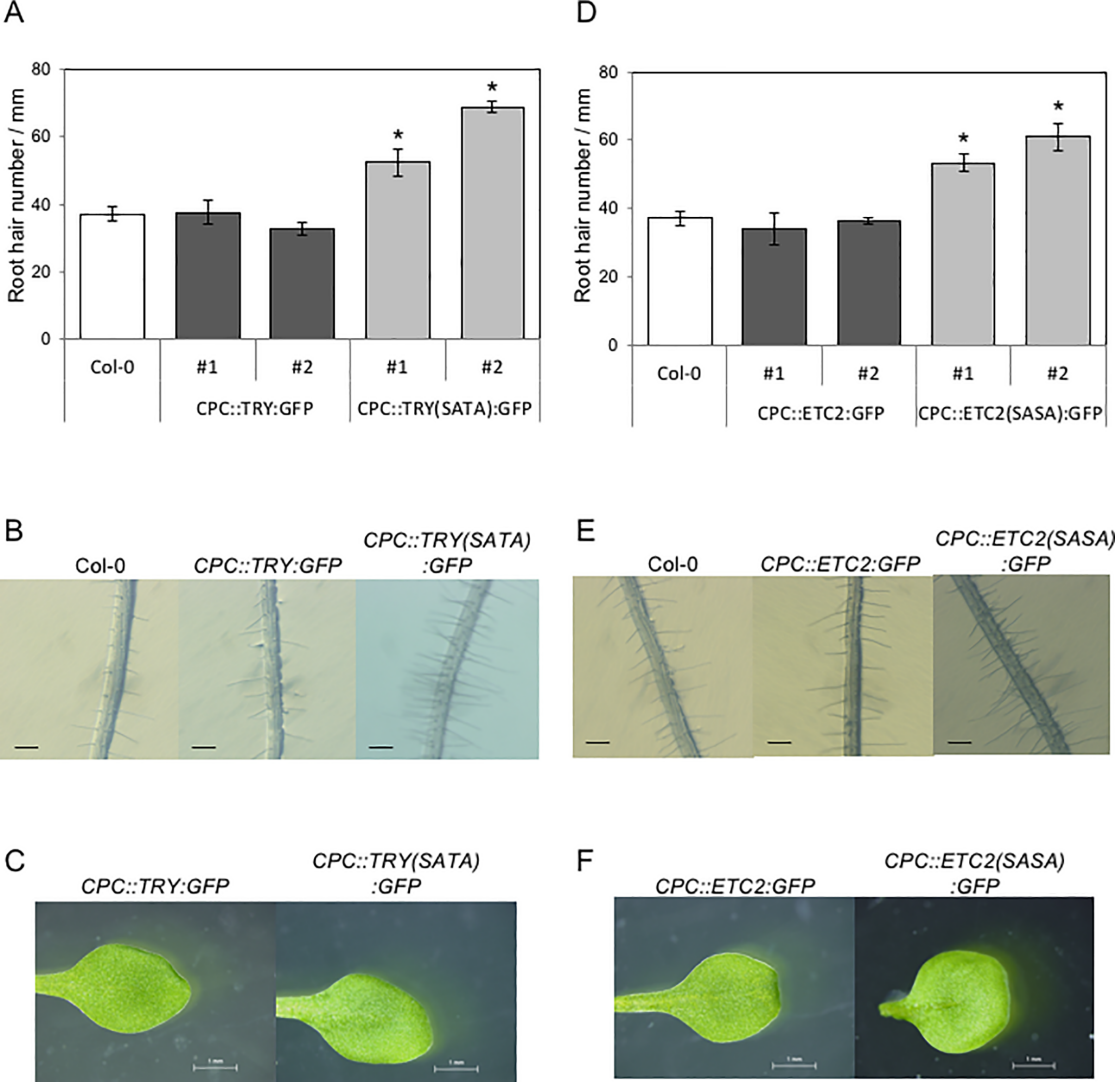
Epidermal phenotypes of transgenic *Arabidopsis* plants expressing C-terminal modified TRY and ETC2. (A) Root hair number in the wild type Col-0, *CPC*::*TRY*:*GFP*, and *CPC*::*TRY(SATA)*:*GFP* transgenic lines. The number of root hairs per millimeter was determined by counting in 10-day-old seedlings (*n* = 5). The error bars indicate standard deviation (SD). Bars marked with asterisks indicate a significant difference between the wild type Col-0 and the transgenic lines (*p* < 0.05). (B) Root hair phenotypes of 10-day-old seedlings in the wild type Col-0 and in the transgenic plants. Scale bars: 200 μm. (C) Phenotypes of trichomes in two-week-old third leaves in the wild type Col-0 and transgenic plants. Scale bars: 500 μm. (D) Root hair number in the wild type Col-0, *CPC*::*ETC2*:*GFP*, and *CPC*::*ETC2(SASA)*:*GFP* transgenic lines. The number of root hairs per millimeter was determined by counting in 10-day-old seedlings (*n* = 5). The error bars indicate standard deviation (SD). Bars marked with asterisks indicate a significant difference between the wild type Col-0 and transgenic lines (*p* < 0.05). (E) Phenotypes of the root hairs of five-day-old seedlings of the wild type Col-0 and transgenic plants. Scale bars: 200 μm. (F) Phenotypes trichomes in two-week-old third leaves the wild type Col-0 and transgenic plants. Scale bars: 500 μm.

The amino acids predicted to be the sites of phosphorylation sites in the C-terminal regions of TRY and ETC2 were substituted by alanine (S2A and T7A in TRY, and S2A and S8A in ETC2) to obtain *CPC*::*TRY(SATA)*:*GFP* and *CPC*::*ETC2(SASA)*:*GFP* transgenic plants (Fig 1C). We analyzed the *CPC*::*TRY*:*GFP* and *CPC*::*TRY(SATA)*:*GFP* transgenic plants to compare the functions of the wild type and mutated TRY (Fig 2A–2C). Consistent with the findings of previous studies, the *CPC*::*TRY*:*GFP* transgenic plants did not induce ectopic root hairs compared with the number of root hairs in the wild-type Col-0 plants (Fig 2A and 2B) [20]. On the contrary, two independent *CPC*::*TRY(SATA)*:*GFP* transgenic lines (#1 and #2) showed a significant increase in the number of root hairs compared with that in the wild type Col-0 plants (Fig 2A and 2B). To compare the functions of ETC2 and those of its amino acid-substituted version, we analyzed the *CPC*::*ETC2*:*GFP* and *CPC*::*ETC2(SASA)*:*GFP* transgenic plants (Fig 2D–2F). Consistent with the findings of previous studies, the *CPC*::*ETC2*:*GFP* transgenic plants did not induce ectopic root hairs compared with the number of root hairs in the wild-type Col-0 plants (Fig 2D and 2E) [20]. In contrast, two independent *CPC*::*ETC2(SASA)*:*GFP* transgenic lines (#1 and #2) showed a significant increase in the number of root hairs compared with that in the wild type Col-0 plants (Fig 2D and 2E). These results suggest that amino acid residues substituted by alanine are important for the function of TRY and ETC2. All the transgenic plants observed in this study showed no-trichome phenotypes, which was consistent with the phenotypes of *CPC*::*TRYΔC*:*GFP* and *CPC*::*ETC2Δ*:*GFP* transgenic plants (Fig 2C and 2F) [20].

### Amino acid substitution in TRY and ETC2 did not disturb their expression levels

**Fig 3:**
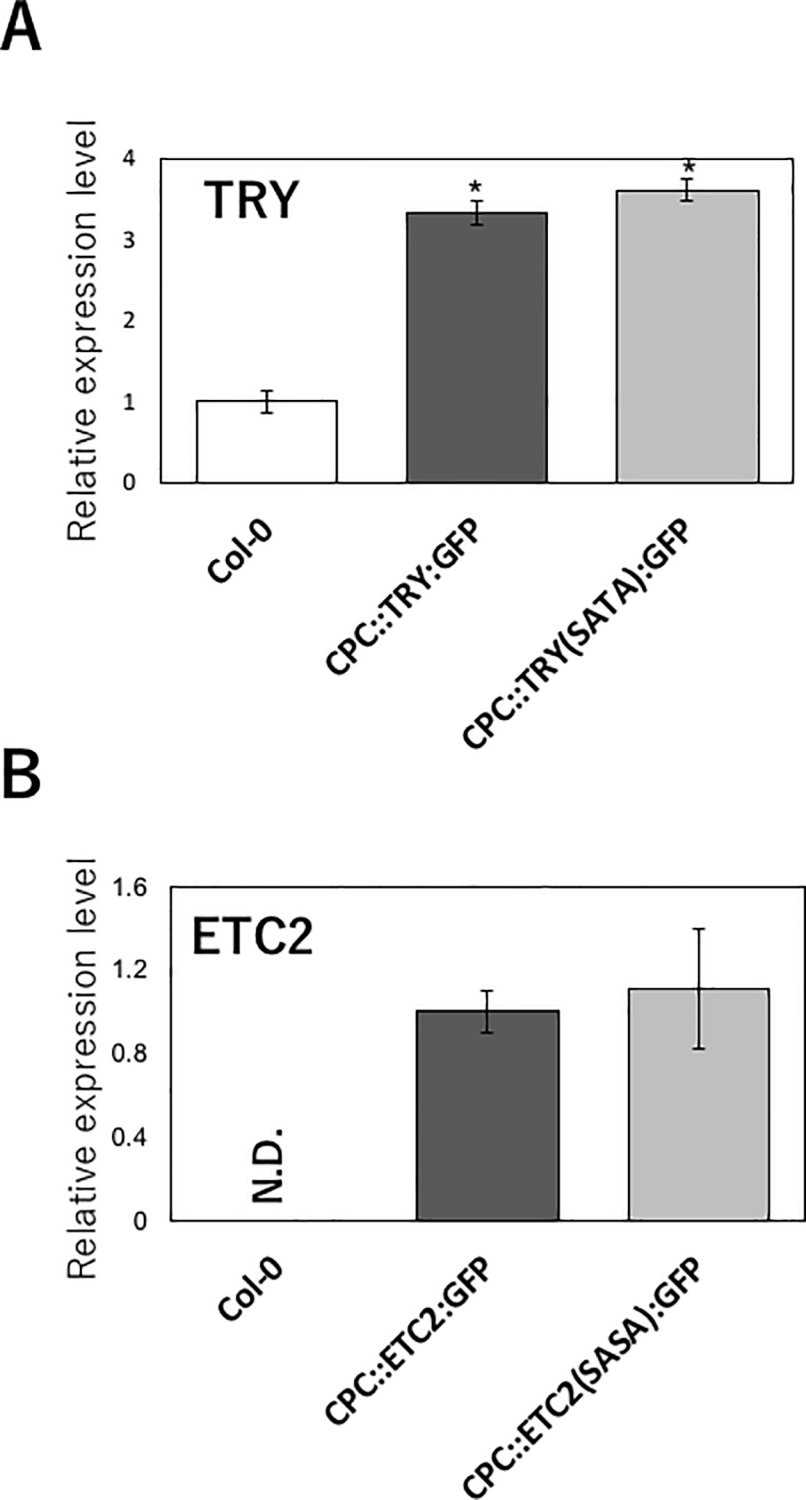
Expression analyses of *TRY* and *ETC2*. (A) Expression analysis of *TRY*. The *TRY* gene expression level in each line was calculated relative to the reference gene *ACT2*, and then normalized to that in the wild type (Col-0) plant, which was set to 1. (B) Expression analysis of *ETC2*. The *ETC2* gene expression level in each line was calculated relative to the reference gene *ACT2*, and then normalized to that in the *CPC*::*ETC2*:*GFP* transgenic plant, which was set to 1. The error bars indicate standard deviation (SD). Bars marked with asterisks indicate significant difference between the wild type Col-0 and transgenic lines (*p* < 0.05). N.D. means not detectable.

To investigate the effect of amino acid substitution in TRY and ETC2 on their transcriptional and post-transcriptional levels, real-time PCR was performed (Fig 3). The relative expression level of *TRY* in the *CPC*::*TRY*:*GFP* and *CPC*::*TRY(SATA)*:*GFP* transgenic plants was approximately three-times higher than that in the wild type Col-0 root tissues (Fig 3A). This demonstrates the actual expression of the introduced *TRY* and *TRY(SATA)* genes in the transgenic plants. There was no substantial difference in the *TRY* mRNA levels between the *CPC*::*TRY*:*GFP* (Fig 3A; dark gray bar) and *CPC*::*TRY(SATA)*:*GFP* (Fig 3A; light gray bar) transgenic plants.

As the ETC2 gene is hardly expressed in roots [15], we could not detect its expression in the wild type Col-0 root tissues (Fig 3B). Similar to that in *TRY*, there was no substantial difference in the *ETC2* mRNA levels between the *CPC*::*ETC2*:*GFP* (Fig 3B; dark gray bar) and *CPC*::*ETC2(SASA)*:*GFP* (Fig 3B; light gray bar) transgenic plants. These results suggest that the amino acid substitutions in TRY and ETC2 did not affect their transcriptional levels.

### Effect of amino acid substitutions on the stability of TRY and ETC2

**Fig 4:**
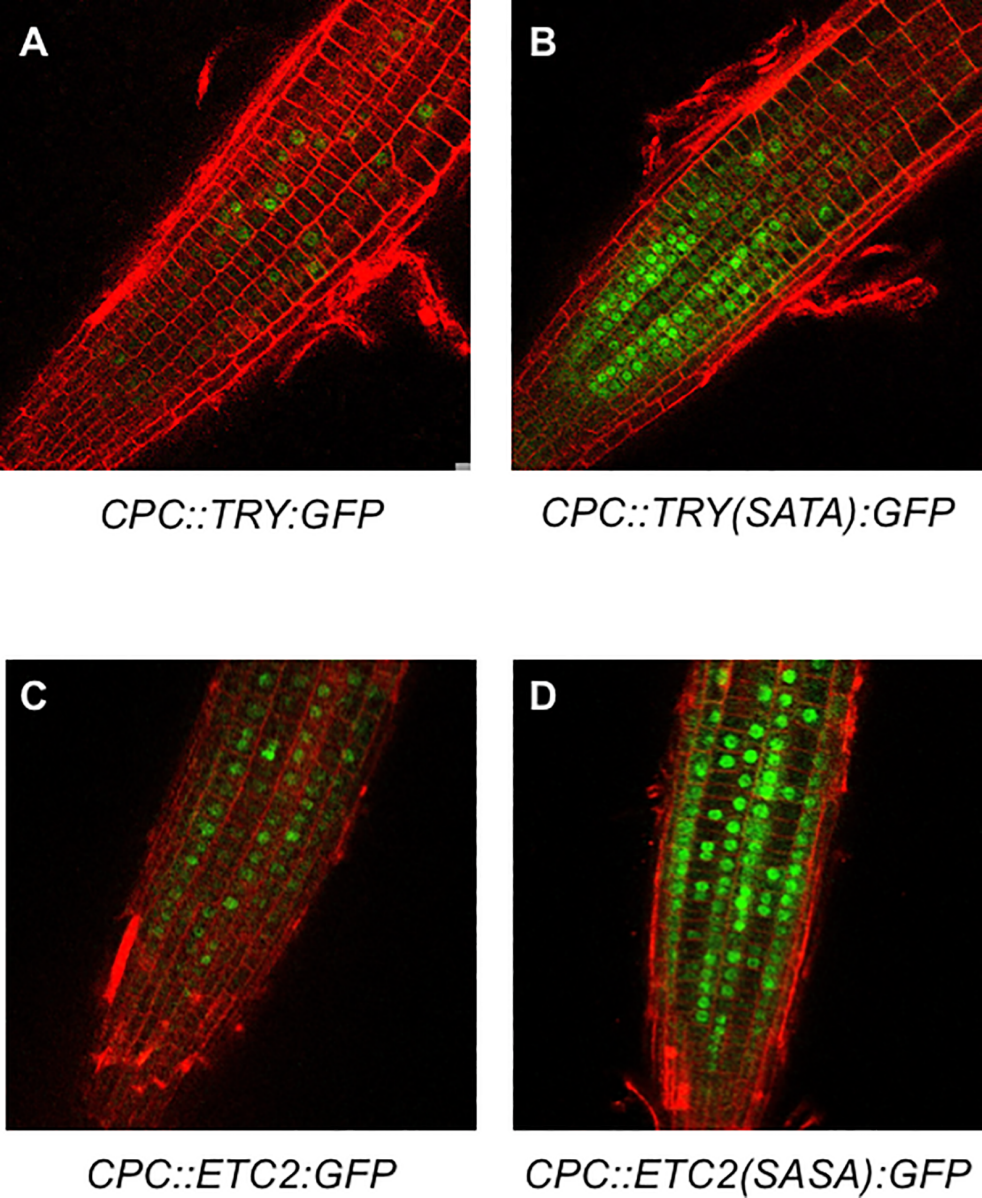
Distribution of GFP fluorescence in *CPC*::*TRY(SATA)*:*GFP* and *CPC*::*ETC2(SASA)*:*GFP* transgenic plants. Confocal laser scanning microscopy images showing GFP (green) and PI (red) fluorescence in the root epidermis of 10-day-old seedlings. The *CPC*::*TRY*:*GFP* (A), *CPC*::*TRY(SATA)*:*GFP* (B), *CPC*::*ETC2*:*GFP* (C), and *CPC*::*ETC2(SASA)*:*GFP* (D) transgenic plants were observed.

Green fluorescent protein fluorescence was observed in the root epidermal cells of *CPC*::*TRY*:*GFP*, *CPC*::*TRY(SATA)*:*GFP*, *CPC*::*ETC2*:*GFP*, and *CPC*::*ETC2(SASA)*:*GFP* transgenic plants (Fig 4). In accordance with the findings of a previous study only weak unclear TRY:GFP protein localization was observed in the *CPC*::*TRY*:*GFP* transgenic plants [20] (Fig 4A). Contrarily, evidently strong GFP fluorescence was observed in the roots of *CPC*::*TRY(SATA)*:*GFP* transgenic plants (Fig 4B). Furthermore, in accordance with the findings of a previous study, only weak unclear ETC2:GFP protein localization was observed in the *CPC*::*ETC2*:*GFP* transgenic plants [20]. In contrast, the roots of *CPC*::*ETC2(SASA)*:*GFP* transgenic plants showed strong GFP fluorescence, similar to that in TRY(SATA):GFP (Fig 4C and 4D). These results suggest that amino acid substitutions in the C-terminal regions of TRY and ETC2 might lead to the accumulation of these proteins in the root epidermis and result in the induction of a large number of root hairs.

**Fig 5:**
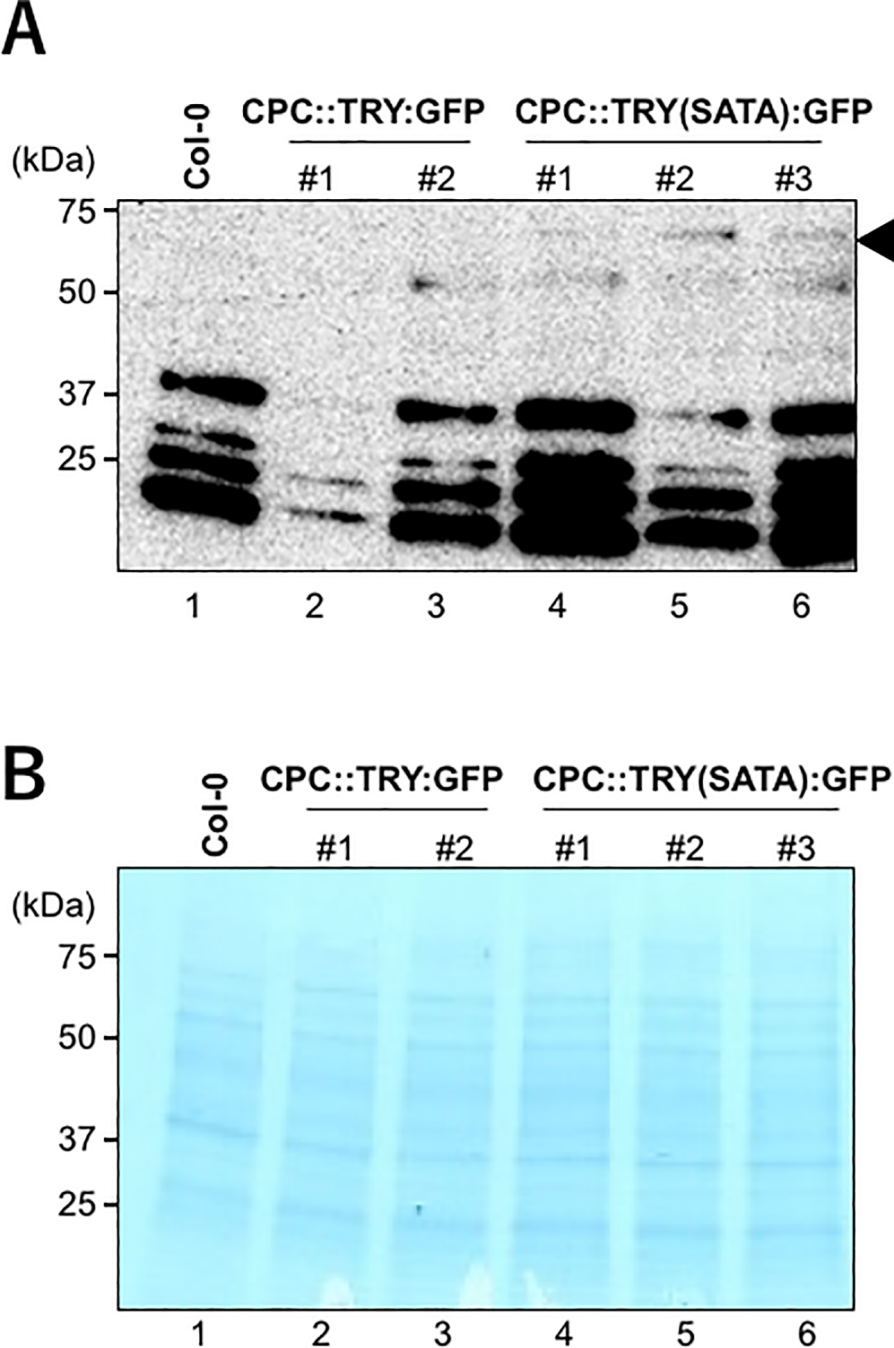
Immunoblot analysis of GFP fusion proteins. (A) Wild-type Col-0 (lane 1), TRY:GFP (lanes 2 and 3), and TRY(SATA):GFP (lanes 4–6) proteins (indicated by the arrowhead) were immunoblotted using anti-GFP antibodies. Molecular weights are shown on the left. (B) Coomassie-stained sample showing equal loading of protein samples (20 μg each lane). Molecular weights are shown on the left.

The apparent disparity in the accumulation levels of TRY:GFP and TRY(SATA):GFP fusion proteins in the root epidermal cells was demonstrated by immunoblot analysis of the proteins extracted from the root tissue of wild-type Col-0, *CPC*::*TRY*:*GFP*, and *CPC*::*TRY(SATA)*:*GFP* transgenic plants using an anti-GFP antibody. In the transgenic plants expressing *CPC*::*TRY(SATA)*:*GFP*, weak bands of the predicted molecular mass (65 kDa) corresponding to TRY(SATA):GFP were detected (indicated by arrowhead) (lanes 4–6; Fig 5). However, bands corresponding to TRY:GFP was not detected in the *CPC*::*TRY*:*GFP* transgenic plants (lanes 2 and 3; Fig 5). These results demonstrate that the substitution of the C-terminal S2 and/or T2 of TRY apparently contributes to the stability of this protein, similar to that observed with the deletion of its C-terminus [20].

## Discussion

Previous studies have shown that the overexpression of five members of CPC family, including CPC, TRY, ETC1, ETC2, and CPL3 (using the CaMV 35S promoter), generates essentially the same epidermal phenotype (ectopic root hairs and reduced trichome production) [10–14]. Therefore, these five single-repeat R3 CPC-like MYB proteins are thought to determine the fate of root hair cells and inhibit the formation of trichome. However, TRY and ETC2 were reported to be less effective in root hair formation and trichome inhibition than those by CPC, ETC1, and CPL3 when introduced under the control of CPC promoter in *Arabidopsis* [20, 29]. Thus, TRY and ETC2 were expected to differ in some functions, compared with those of CPC, ETC1, and CPL3. The TRY and ETC2 proteins have longer C-terminal regions than those of CPC, ETC1, and CPL3. In this study, we focused on the C-terminal regions of TRY and ETC2, to identify the amino acids critical for their specific ability.

We generated amino acid substituted TRY and ETC2 constructs (Fig 1C). Two amino acids in the C-terminal regions of TRY and ETC2 were substituted by alanine, respectively (Fig 1C). Both the amino acid-substituted versions of TRY and ETC2 [TRY(SATA) and ETC2(SASA)] significantly induced root hair formation compared with that of their respective control versions (TRY and ETC2; Fig 2A, 2B, 2D and 2E). These results suggest that serine and/or threonine in the C-terminal region of TRY (S2 and T7; Fig 1C) and serine in the C-terminal region of ETC2 (S2 and S8; Fig 1C) are important amino acid(s) for their root hair inducing activity. These amino acids (S2 and T7 in TRY C-terminal region, and S2 and S8 in ETC2 C-terminal region) are expected to be phosphorylated in plant cells (Fig 1B; S1 Fig). Therefore, it is suggested that phosphorylation might be involved in the specific activity of TRY and ETC2. However, we cannot rule out the possibility that amino acid substitutions might affect protein conformation or aspects other than phosphorylation status. Contrary to the root hair phenotypes, all the transgenic lines observed in this study lacked trichomes on the leaf surface (Fig 2C and 2F), similar to that observed with the deletion of their C-terminal region [20]. These amino acid substitutions might be irrelevant to their function in inhibiting the formation of trichomes on the leaves. The functions of TRY and ETC2 might be different in the roots and leaves. Another possibility is that protein stability might be necessary for function in the roots, but rapid protein degradation might not interfere their ability in the leaves.

By substituting the two amino acids in TRY and ETC2, the promotion of root hair formation was recovered. Whether one of these two amino acids or both are important remains to be examined. Amino acid substitutions did not affect the gene expression levels, similar to that observed with the deletion of C-terminus [20]. This confirms that the functions of TRY and ETC2 are regulated at the post-transcriptional level, and not at the transcriptional level.

In this study, we demonstrated that amino acid substitutions increase the stability of the TRY and ETC2 proteins. However, we could not detect the expected levels of TRY(SATA):GFP by immunoblot analysis (Fig 5). Besides the substituted amino acids, there may be other important amino acids. Further investigations, by examining the effects of the substitution of other amino acids, protein conformation, phosphorylation, and ubiquitination, are needed to obtain a better overall picture of the role of TRY and ETC2 in epidermal cell differentiation.

## Supporting information

S1 Fig Phosphorylation candidate sites of TRY and ETC2 C-terminal regions. Phosphorylation candidate sites of TRY and ETC2 predicted using PhosPhAt (http://phosphat.uni-hohenheim.de/).
(TIFF)
