# Supplementary figures and images for: Amino acid substitutions in CPC-LIKE MYB reveal residues important for protein stability in Arabidopsis roots
Source: PLoS One. 2018 Oct 11;13(10):e0205522. doi: 10.1371/journal.pone.0205522 (PMC6181395; doi:10.1371/journal.pone.0205522)

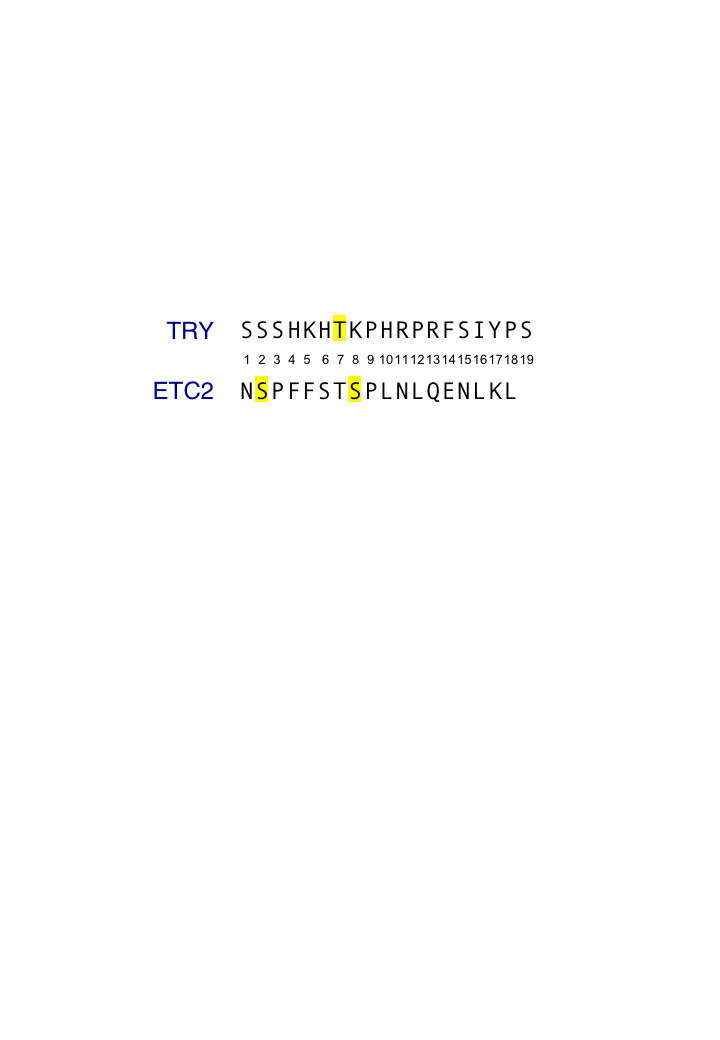

Supplement: S1 Fig — Phosphorylation candidate sites of TRY and ETC2 predicted using PhosPhAt (http://phosphat.uni-hohenheim.de/). (TIFF) [file pone.0205522.s001.tiff]
